# Supplementary material for: The macroevolution of size and complexity in insect male genitalia
Source: PeerJ. 2016 Apr 14;4:e1882. doi: 10.7717/peerj.1882 (PMC4841225; doi:10.7717/peerj.1882)
Supplement: Supplemental Information 1 [file peerj-04-1882-s003.pdf]

The macroevolution of size and complexity in insect male genitalia

Andrey Rudoy and Ignacio Ribera

Institute of Evolutionary Biology (CSIC-Universitat Pompeu Fabra), Passeig Maritim de la Barceloneta 37-49, 08003 Barcelona, Spain

Electronic Supplemental Information

Table S1. Specimens used, with locality, collector, voucher, species group according to Rudoy et al. (in press) and genbank accession numbers. Nf and Nm, number of females and males measured respectively; Ng, number of male genitalias measured; lf and lm, length of females and males respectively; stv.lf and stv.lm, standard deviation of lf and lm (for less than seven specimens the total range is given); lg, length of the male genitalia; stv.lg, standard deviation of lg.

Table S2. Reconstructed values of the measured variables in all branches of the phylogeny (see Figure S2 for the location of the branch numbers). The raw values of the initial and final nodes of each branch are given, together with the amount of phenotypic change, the absolute phenotypic change, and the change measured in darwins (x10^6). In pink, composite branch after deleting terminals with missing males. In yellow, reconstructed values.

Figure S1. Aedeagus of *L. gerhardti*, with A) maximum length (*lg*); B) perimeter, outlined by hand and measured in ImageJ; and C) log-log plot of the regression between the scale of the box and the number of boxes necessary to cover the image, obtained with the Fractal Dimension Estimator (see Methods). The absolute value of the slope of the regression line is the fractal dimension of the image (in this case, the outline of the aedeagus).

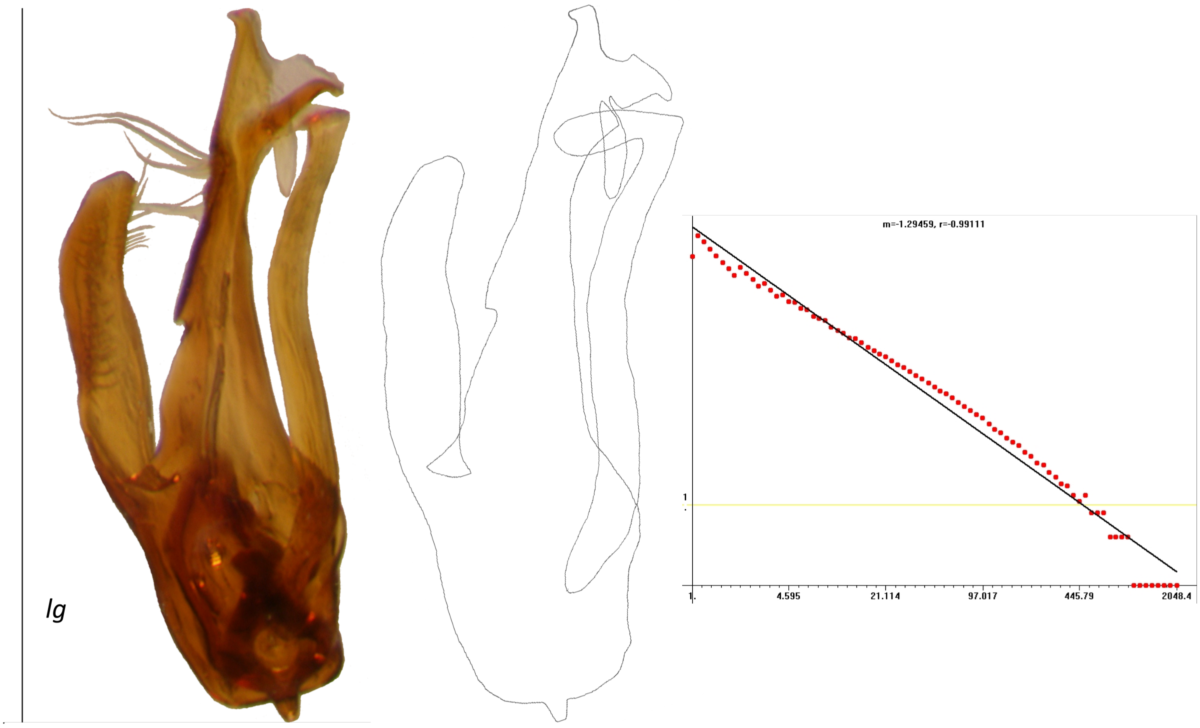

A B C

Figure S2. Phylogeny of *Limnebius*, with the branch numbers used in Table S2.

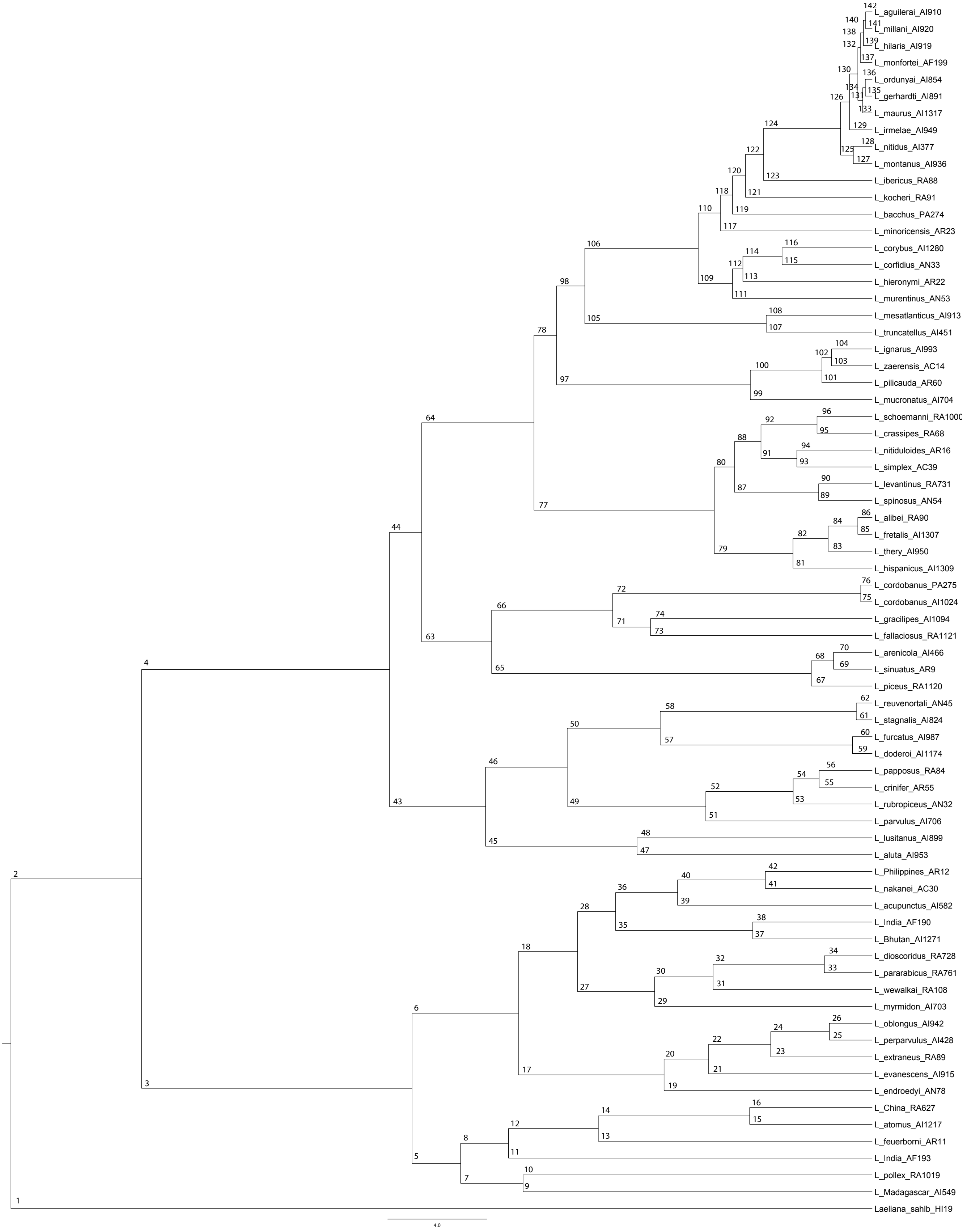

Figure S3. Kernel density estimates (KDE) of the probability density function of the PAD measurements and the branch lengths, for all and the terminal branches; and for the subgenera *Bilimneus* and *Limnebius* s.str. KDEs were used for illustrative purposes only; see Table 4 for the parameters of the comparison between the average and variance of the different variables.

A) Branch lengths and phenotypic change, terminals vs all branches

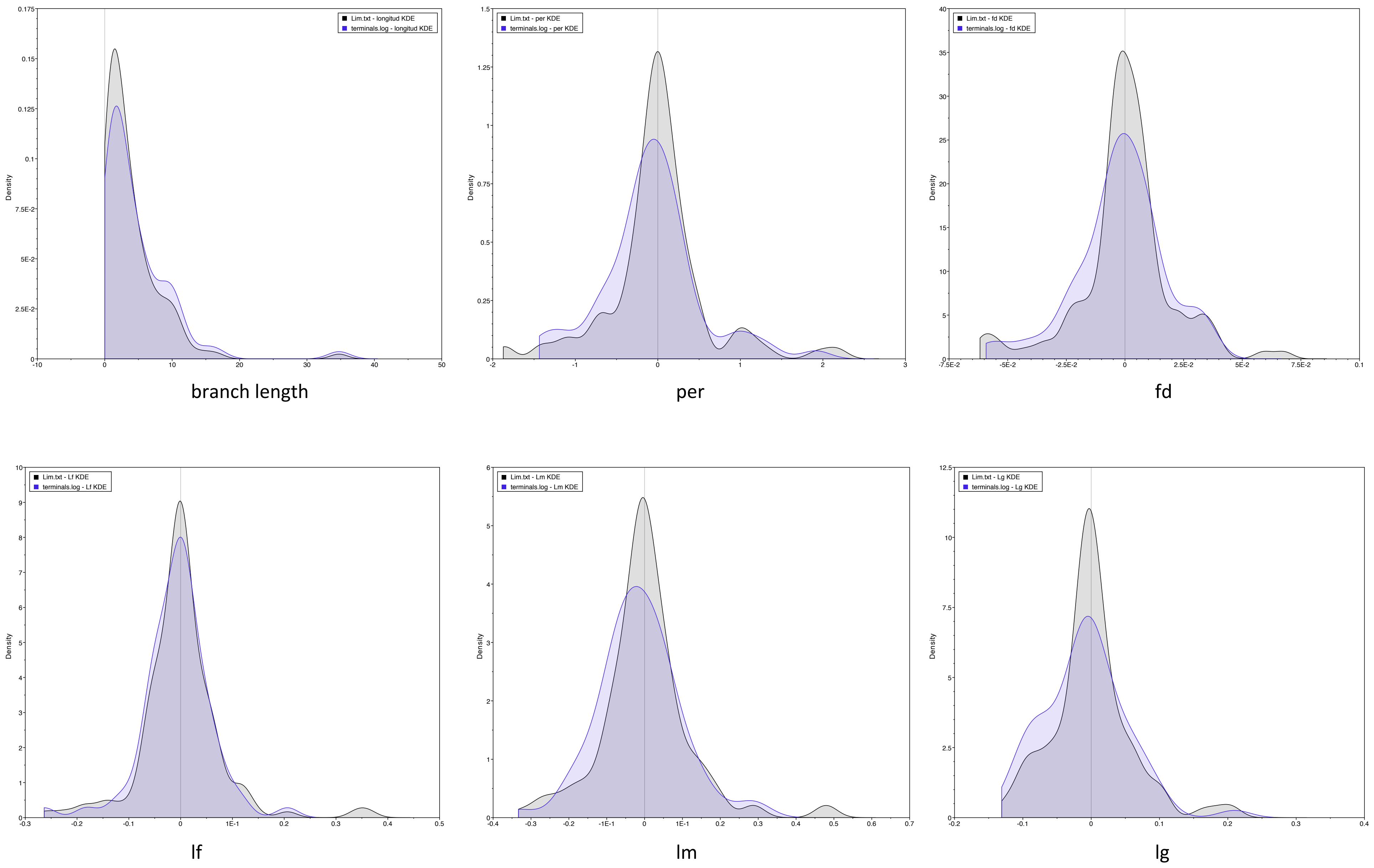

B) Branch lengths and phenotypic change, *Bilimneus* vs *Limnebius* s.str.

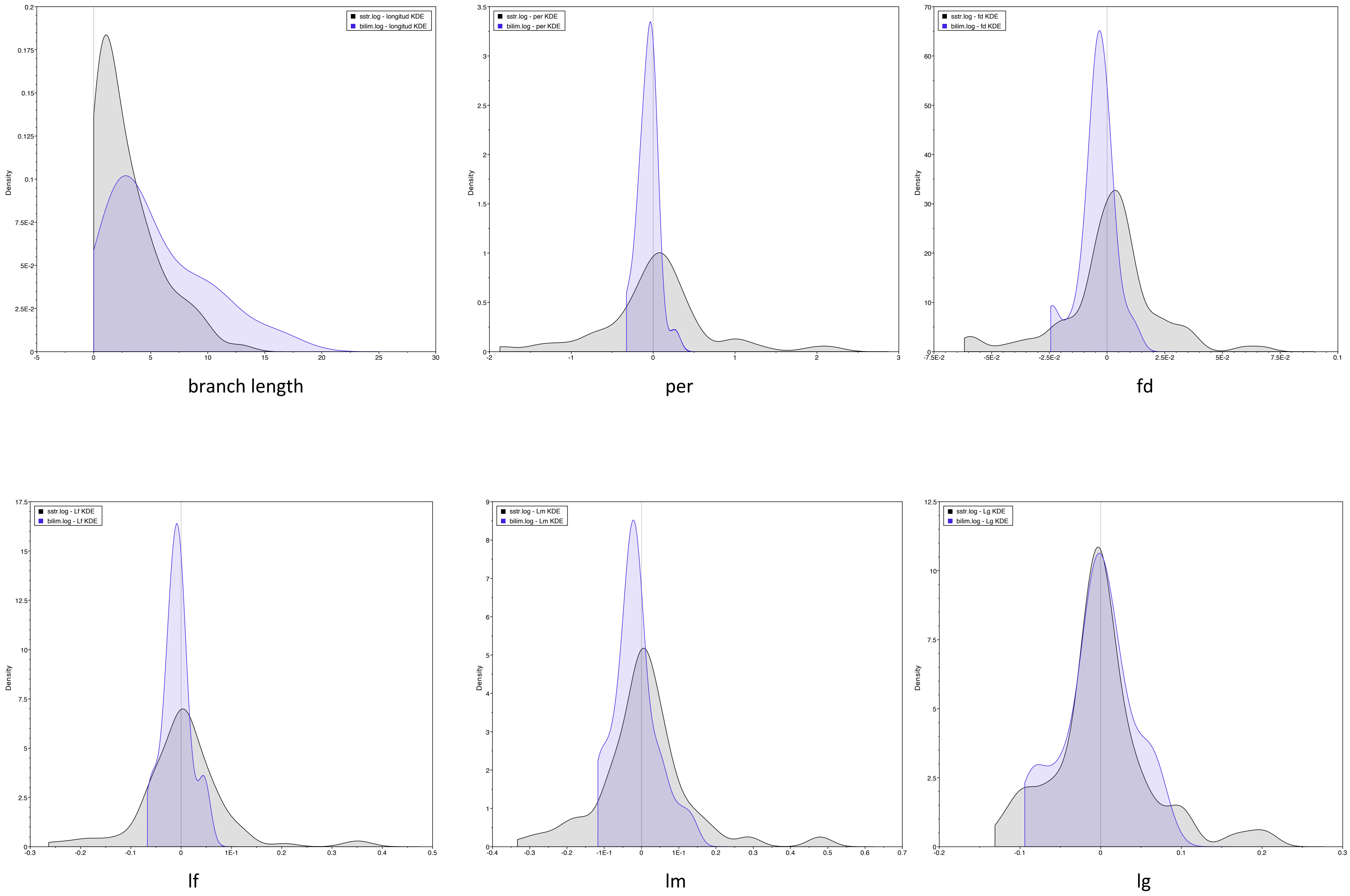

C) darwins, terminals vs all branches

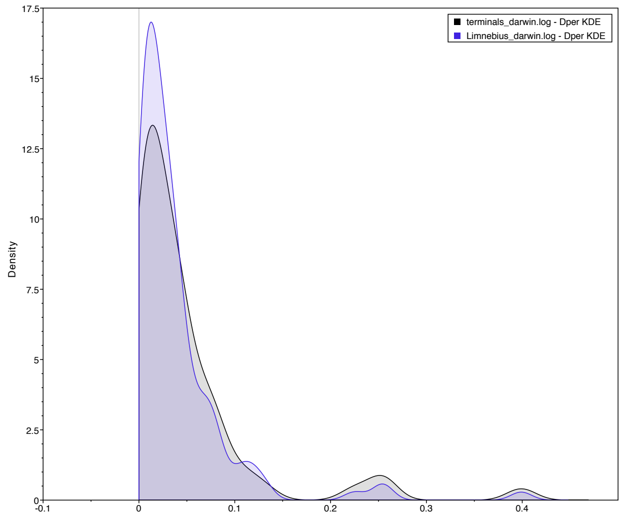

per

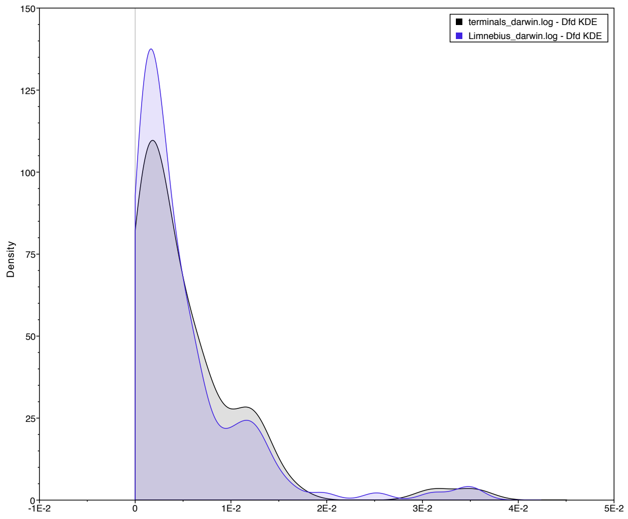

fd

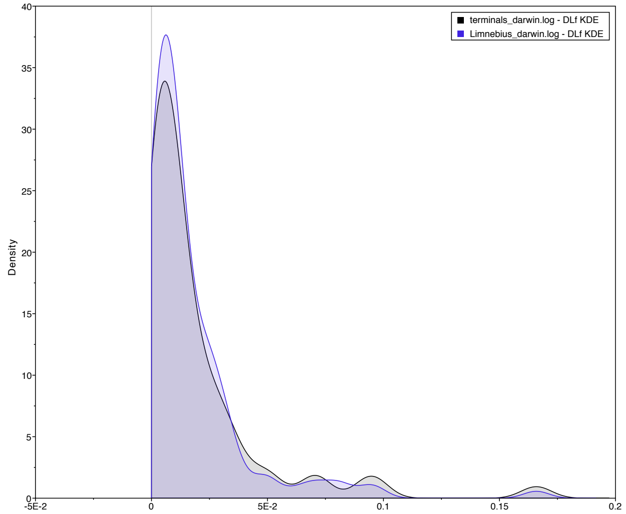

lf

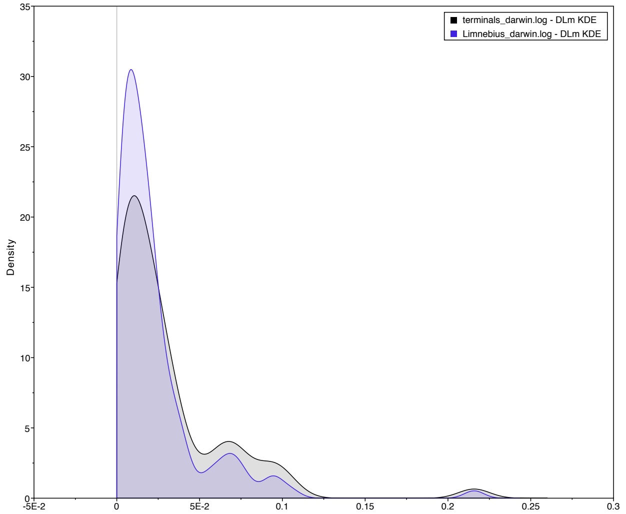

lm

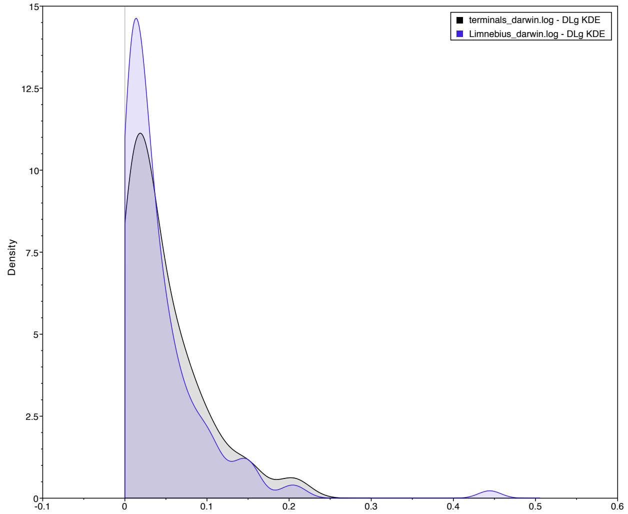

lg

D) darwins, *Bilimneus* vs *Limnebius* s.s.tr.

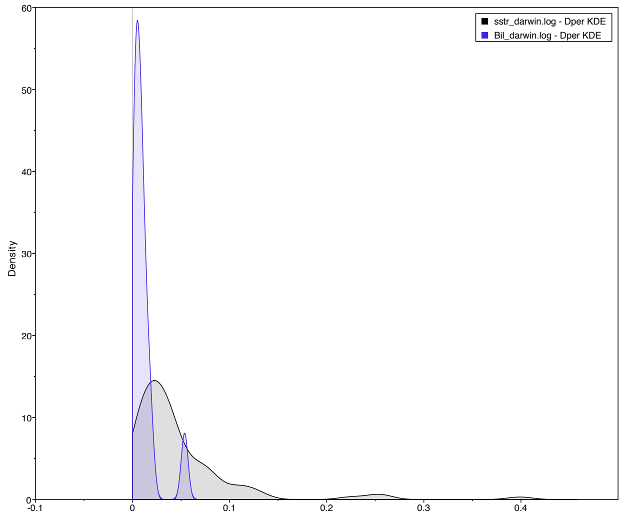

per

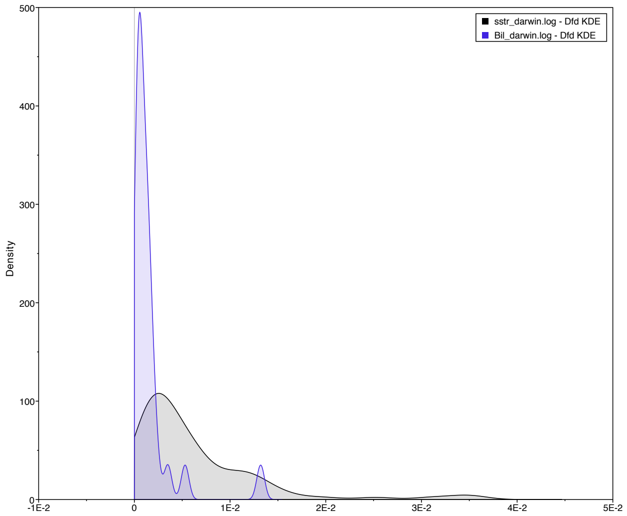

fd

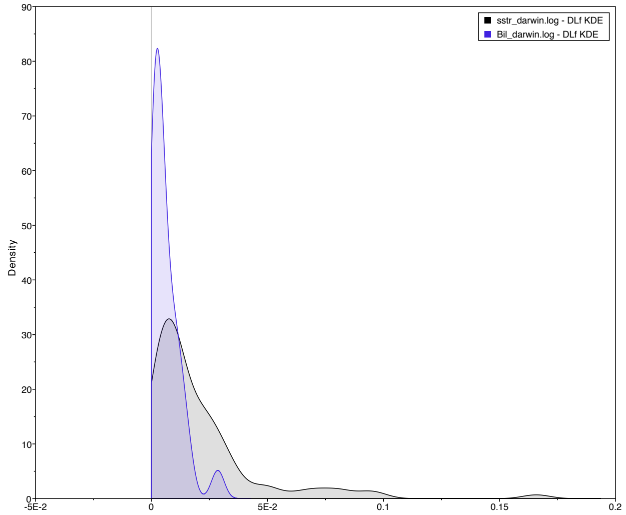

lf

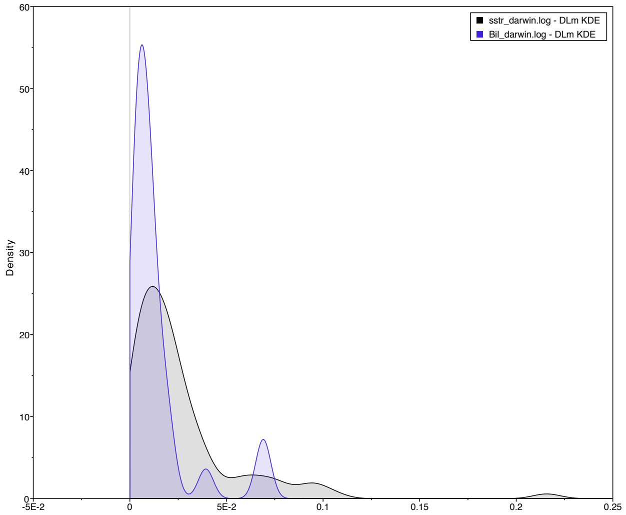

lm

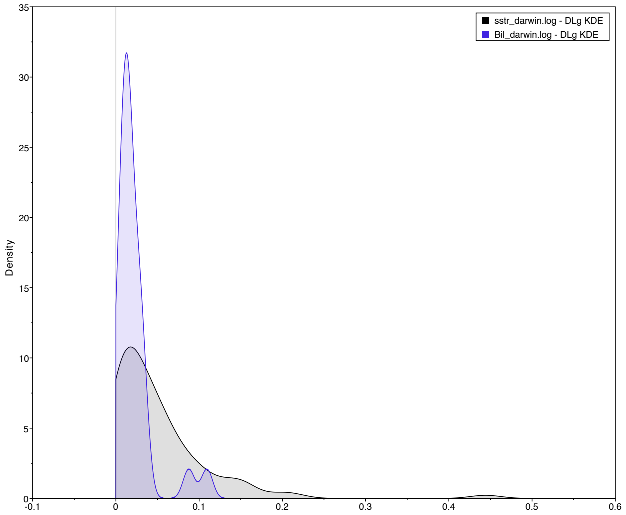

lg
